# The lived experiences of South African parents during high-risk pregnancy and prolonged neonatal intensive care unit admission

**DOI:** 10.4102/hsag.v31i0.3342

**Published:** 2026-08-12

**Authors:** Linda H. Goldberg, Jacqueline K. Bezuidenhout, Janine van der Linde

**Affiliations:** 1 Department of Paediatrics, School of Clinical Medicine, Faculty of Health Sciences, University of the Witwatersrand, Johannesburg, South Africa; 2 Department of Occupational Therapy, School of Therapeutic Sciences, Faculty of Health Sciences, University of the Witwatersrand, Johannesburg, South Africa

**Keywords:** prematurity, NICU, parental lived experience of the parents, psychosocial well-being, private hospitals

## Abstract

**Background:**

Becoming a parent is a significant life event; however, premature birth abruptly alters this transition, creating short- and long-term consequences for both parents and infants.

**Aim:**

This study sought to explore the lived experiences of South African parents throughout the trajectory of premature birth and the subsequent hospitalisation of their baby in the neonatal intensive care unit (NICU).

**Setting:**

The research was conducted in Johannesburg, South Africa, within the private healthcare setting.

**Methods:**

A quantitative, exploratory and descriptive phenomenological study design was used. Purposive sampling identified participants, and 11 interviews were conducted with eight mothers and three fathers. Inductive thematic analysis using MAXQDA identified emerging codes, categories and themes.

**Results:**

Three themes emerged: the journey to the NICU and the pregnancy context, transitioning to parenthood within the unnatural NICU environment, and the emotional journey during the prolonged admission. Parents reported limited psychosocial support from doctors, nurses and other parents.

**Conclusion:**

The challenges experienced by the parents mirrored common themes identified in global research. Parents described the psychosocial challenges during the pregnancy and neonatal admission, and the difficulties of achieving the parental role within the NICU environment. Confounding this was the limited exposure to formal psychosocial counselling by trained professionals.

**Contribution:**

This research highlights the psychosocial challenges experienced by parents during the admission period in private-sector NICUs and the need for increased, planned and structured psychosocial support.

## Introduction

Preterm birth of an infant and subsequent admission to a neonatal intensive care unit (NICU) are widely reported to be highly traumatic experiences for parents (Malouf et al. 2022; McKeown et al. 2023). The fragile medical status of the baby, extended hospital admission periods, and possible uncertain developmental trajectory frequently disrupt the transition to the parental role and have significant implications for parents’ psychological well-being (Siva et al. 2024). Existing research highlights the experiences of post-traumatic stress disorder, acute stress disorder, anxiety and depression during admission to the NICU (McKeown et al. 2023).

Although psychological distress and difficulties in assuming the parental role are consistently observed in all contexts, they vary according to individual resources, socio-economic conditions and maternal–paternal differences (Marie et al. 2025). Psychological distress could be influenced by the physical environment of the NICU, the medical fragility of the baby, separation from their baby and challenges with the staff (Siva et al. 2024; Van Wyk et al. 2024).

The World Health Organization (WHO) defines a preterm birth as any live birth before 37 completed gestational weeks or fewer than 259 days from the first date of the last menstrual cycle (WHO 2023). Prematurity and its complications account for the most common cause of mortality in children under 5 years, and in 2020, the WHO estimated that 10% – 15% of babies in South Africa were born prematurely (WHO 2023).

Both intrinsic and extrinsic factors influence parental perceptions and experiences of their baby’s premature birth in the NICU. Intrinsically, parents feel a strong sense of separation and constrained opportunities to participate in parenting roles (Citter & Ghanouni 2021). Extrinsically, the physical environment of the NICU, along with the appearance of their infant and behaviour patterns, can lead to negative experiences (Van Wyk et al. 2024). Physical distance and reduced opportunities to participate in daily care routines create a sense of physical and emotional separation, leading parents to feel like visitors rather than parents (Van Wyk et al. 2024). Importantly, both the bonding process and the creation of appropriate parent-baby attachment are affected by the NICU environment (Ettenberger et al. 2021; Kim et al. 2020). Therefore, the psychosocial discord that accompanies admission and post-discharge NICU admission may have a long-lasting impact on both parents and the baby.

Globally, there has been a shift in the focus of care within the NICU, moving away from the traditionally dominant medical care-driven model towards Family Centred Care (FCC) (Reid, Bredemeyer & Chiarella 2021). The American Academy of Paediatrics began emphasising the importance of FCC in 2003, further endorsing its implementation in 2012 (Committee on Hospital Care and Institute for Patient- and Family-Centered Care 2012). With the introduction of the FCC approach, medical fragility and the care of the preterm baby are no longer the sole priority of the medical fraternity within the NICU (Reid et al. 2021). The FCC philosophy emphasises the need for healthcare professionals to understand and consider parental experiences in the NICU to create an environment where parents are supported and partner with the medical team in infant care (Abukari & Schmollgruber 2023).

The link between FCC and improved emotional support and positive emotional experiences for parents in the NICU has been described in the literature (Abukari & Schmollgruber 2023). One of the pillars of FCC, the promotion of information sharing, has been shown to reduce stress, anxiety and depression amongst parents in the NICU (Abukari & Schmollgruber 2023).

Embracing the essence of FCC, ‘Best Practice Guidelines’ for neurodevelopmental supportive care in the NICU were developed and introduced in South Africa in 2010 (Lubbe 2010). However, barriers related to policies, staff shortages, education and patient load continue to impact the implementation of the South African ‘Best Practice Guidelines’ in NICUs (Malepe, Havenga & Mabusela 2022). The South African neonatal care landscape is characterised by the contrast of the overburdened public healthcare sector and the better-resourced private healthcare sector (Ballot et al. 2019).

Research exploring the lived experiences of the journey of the South African parents in NICU in both the public and private healthcare sectors is limited (Steyn, Poggenpoel & Myburgh 2017). Research from the public health sector has highlighted the importance of cultural beliefs and practices of parents in the NICU, the challenges of infant care after discharge from the NICU, and the challenges of decision-making, counselling and communication with parents in the NICU environment (Buys & Gerber 2021; Nyaloko et al. 2024).

Within the private healthcare sector, the literature is sparse, with a single study exploring the lived experiences of parents at a Johannesburg-based NICU (Steyn et al. 2017). Although there is substantial global literature on parental experiences of the psychosocial support needed in the NICU, the unique perspectives of South African parents, particularly in the private healthcare sector, warrant focused attention. The introduction of the FCC has improved understanding and consideration of parents’ NICU experience, creating an environment in which parents are supported and partnered in infant care (Yeomans et al. 2026).

The study was designed to explore the parental experiences of their pregnancy, premature birth of their baby, and the consequent admission to the NICU, to identify common themes emerging in different private hospital settings and to compare them with universal research findings. By identifying both positive and negative experiences of parents, holistic and multidisciplinary care can be provided to infants and parents during this stressful time (Reid et al. 2021).

## Research methods and design

### Study setting

The study was carried out in Johannesburg, an urban setting in South Africa. The private sector of Johannesburg has numerous hospitals with NICU facilities and specialist obstetric and foetal-maternal medical services, making it an appropriate setting for study.

The decision to focus on multiple private NICU settings, rather than on a single facility, was deliberate. Only one previously published study has explored the lived experiences of parents in a private NICU in Johannesburg (Steyn et al. 2017); therefore, this research aimed to extend and contextualise these findings in a broader range of private healthcare settings. The four neonatal facilities featured in this study were not handpicked; they emerged from the sampling process as the sites where eligible participants had received care. All four were in the private healthcare sector, meaning the families involved relied on medical aid to cover the costs of their infants’ NICU stays.

It is important to note that the lived experiences described in this study are specific to the private healthcare context and are not automatically transferable to public-sector NICU settings in Johannesburg or elsewhere in South Africa, where resource constraints, patient volumes and staffing ratios differ considerably.

### Design

A qualitative, exploratory and descriptive phenomenological design was used to describe the lived experiences of parents during high-risk pregnancy and prolonged admission to the NICU (Im et al. 2023). Understanding what parents experienced during this time, not just whether they struggled, but what that struggle felt like, fit with a descriptive phenomenological approach (Mash et al. 2025). This allowed parents to tell their stories in their own words, rather than the researcher’s active interpretation of what an experience means. Qualitative research uses interviews as its primary data collection method (Mash et al. 2025). The interview process, through semi-structured interviews, allowed participants to narrate the experiences of their pregnancies, the premature birth of their child, and subsequent admission to the NICU, and how these experiences affected them. Inductive thematic analysis ensured that the findings emerged from participants’ statements rather than from the researcher’s expectations.

### Population and sampling

The study population comprised parents of premature infants discharged from private hospital NICUs in Johannesburg. Healthcare professionals (physiotherapists and occupational therapists) working with premature infants after NICU discharge assisted in providing information to potentially eligible participants.

Parents were informed about the study and that their participation was voluntary and would not affect ongoing therapy or healthcare services. Parents who expressed interest in participating provided consent for their contact details to be shared with the researcher, thereby protecting personal information and complying with the *Protection of Personal Information Act (POPIA)*. Subsequently, direct contact was made, and further informed consent to participate was obtained. This sequence meant that by the time the researcher and participant spoke, participation was already a freely made choice rather than something that felt expected or obligatory. During recruitment, no intentional demographic restrictions were applied, and participants were recruited based on eligibility criteria rather than demographic characteristics. Recruiting across multiple therapy practices and four different NICU facilities naturally introduced variation in parental background, distances travelled, visiting experiences, and levels of support received. However, despite attempts to recruit broadly, the final cohort of participants reflected limited demographic diversity, which may be attributed to the private healthcare context and the voluntary nature of participation.

The use of healthcare professionals as gatekeepers was a deliberate decision based on ethical and practical considerations. These professionals knew the families, understood the sensitivity of their experiences, and were well placed to introduce the study without pressure or coercion by preventing the researcher from engaging in direct recruitment. It also reduced potential selection bias by ensuring that recruitment was guided by predefined eligibility criteria rather than by the researcher’s preferences. The unit of recruitment was always the parent, not the institution. No hospitals were approached, shortlisted or formally recruited as research sites. Instead, they spontaneously emerged as the places where eligible participants had recently received care.

Inclusion criteria included gestational age less than 37 weeks at birth; admission to a private hospital NICU in Johannesburg for at least 4 weeks; and discharge home less than 8 months before the interview. Exclusion criteria included any pre-existing therapeutic relationship with the researcher in her capacity as a physiotherapist.

For this study, criterion-based purposive sampling was employed to ensure that only parents who had undergone a high-risk pregnancy, experienced prolonged NICU admission of their premature infant, and could still recall the experience clearly enough to describe it meaningfully.

This approach aligns with the lived experience criterion central to phenomenology, privileging depth of insight over demographic representativeness. By selecting participants who had personally navigated the challenges of NICU admission, the study elicited rich, nuanced accounts of emotional, social and contextual realities.

### Data collection

The interview guide was developed in line with the descriptive phenomenological design, using three broad open-ended questions and flexible prompts to elicit rich, participant-centred narratives of parents’ lived experiences without imposing researcher interpretation. The primary researcher conducted the interviews between January 2023 and July 2023. The interviews were conducted in English, as it was the shared language between the participants. The duration varied considerably, ranging from 15 min to just over an hour. The duration of the interviews varied according to how the participants communicated. Some parents were naturally concise; others needed time to find the words for experiences they had never spoken about in a formal setting.

Of the 11 interviews, 10 were conducted at the parents’ residence, with a single interview, according to the parent’s preference, conducted in a coffee shop of their choice. The interview consisted of three semi-structured open-ended questions: (1) *describe your family support system*; (2) *describe the pregnancy and birth, including parental expectations*; and (3) *describe your experiences of NICU admission*. These questions guided the researcher in initiating conversations, but additional prompts were used to uncover more information about the participants, their experiences of pregnancy and delivery, and their admission to the NICU. The first two questions aimed to establish pre-existing support systems and identify stressors before NICU admission that could influence their NICU experiences. This added richness to the data gathered on the parents’ lived experiences.

The interviews were recorded and transcribed verbatim using voice-to-text technology, and peer debriefing was conducted. To ensure reflexivity and protect against preconceived biases, the researcher kept field notes to capture contextual and non-verbal data during interviews and a reflective journal throughout both data collection and analysis, regularly documenting her thoughts and emotions. The research supervisors conducted reviews and analyses of voice recordings and transcriptions to prevent bias. The recordings were downloaded immediately after each interview and transferred to the secure platform without delay.

Upon completion of the interview, participants completed a demographic questionnaire from which parental and contextual information was obtained. Information included the age of the parents, gender, occupation, education level, employment status, pregnancy characteristics (planned pregnancy, assisted pregnancy, multiple pregnancy and any maternal health conditions). Contextual information highlighted aspects of the NICU environment, such as distance from home, visiting hours, accommodation availability, and staff orientation or written information provided. Data saturation was not predetermined by a specified number of interviews; it was monitored iteratively as data collection progressed. Data saturation was achieved after the 11th interview, when participants consistently expressed recurrent patterns and themes that were consistently replicated rather than extended. Recruitment was closed at this point, ensuring sufficient information power to address the research question.

### Data analysis

Data analysis followed the six phases of reflexive thematic analysis as described by Braun and Clarke (2006).

The researcher conducted an inductive thematic analysis to generate codes and themes to analyse the transcribed interviews. These phases included familiarisation with the data, the generation of initial codes, the identification of emerging themes, the review of the themes, and finally the definition and naming of the themes.

The research supervisors conducted a secondary independent analysis using MAXQDA (VERBI Software 2024), selected for its ability to systematically organise transcripts, support independent coding and maintain a transparent audit trail. Supervisors coded the transcripts without reference to the primary researcher’s framework and compared their emerging codes and themes during structured debriefing sessions. Divergent interpretations were discussed until consensus was reached, resulting in three overarching themes. By facilitating analyst triangulation and revealing blind spots in the primary analysis, MAXQDA strengthened the credibility and trustworthiness of the findings.

### Trustworthiness

Trustworthiness was ensured using the criteria of credibility, dependability, confirmability and transferability as described by Guba and Lincoln (as cited in Ahmed 2024; Enworo 2023). Credibility was enhanced through sustained engagement with participants during data collection, the use of audio-recorded semi-structured interviews, and the maintenance of field notes capturing contextual and non-verbal cues. Peer debriefing was conducted throughout the research process to critically review emerging findings and interpretations.

Dependability was ensured through a transparent description of the research process and the maintenance of an audit trail, including interview transcripts, coding decisions, field notes and analytic memos.

Confirmability was strengthened through reflexive journaling and peer debriefing, which supported the identification and management of potential researcher bias during data collection and analysis. Transferability was supported through thick, rich descriptions of the study context, participants and research procedures to enable readers to assess applicability to similar settings.

### Ethical considerations

Ethical clearance to conduct this study was obtained from the Human Research Ethics Committee (Medical), University of the Witwatersrand (No. M211152).

All *POPIA* regulations regarding the transfer of personal information were complied with. Informed consent was treated as a process rather than a single event. When healthcare professional gatekeepers introduced the study to potentially eligible parents, they provided written information sheets that explained the study’s purpose and what participation would entail. Parents who wished to take the next step consented to sharing their contact details with the researcher; only then was direct contact made. Consent was never assumed and was obtained from both parents for a face-to-face interview, its recording and its transcription. Parents were free to withdraw from the study at any time, even without showing a reason. Confidentiality was protected at all stages of the research. Participants were assigned pseudonyms from the beginning, and no identification information was retained in any part of the analytical data set or research output.

All interviews were recorded with participants’ consent, transferred immediately to a password-protected device, and uploaded to a secure cloud platform accessible only to the research team. Raw data were not shared beyond the research team, and no data were stored on unsecured or publicly accessible platforms at any point. According to the University’s research governance requirements, all data, including recordings, transcripts, field notes and analytical outputs, will be retained for a minimum of 2 years and a maximum of 6 years, after which they will be permanently and securely deleted. The research team was aware that parents came from different cultural, linguistic and religious backgrounds, each carrying their own understanding of pregnancy, premature birth, infant vulnerability and the appropriate role of medical institutions in family life. The researcher approached every interview without assumptions about how a participant’s background shaped their experience, allowing cultural meaning to emerge from the participant’s own narrative. A distress protocol was in place in case of emotional distress experienced by the participants. This protocol included referral numbers for qualified social workers, if required.

## Results

The study explored the lived experiences of parents following the prolonged hospitalisation in the NICU of their premature babies within the private South African healthcare sector.

All interviews were conducted within 6 months after discharge from the NICU.

### Demographic information

Demographic information was collected from the demographic questionnaire, and the results are captured in Table 1.

**TABLE 1:** Parental and pregnancy data (*N* = 11).

Participant	Age (years)	Sex	Education completed	Employed	Singleton or Twins	GA (weeks)	NICU time (weeks)
1	30	F	Tertiary masters	Yes	T	33	6
2	27	F	Tertiary	No	S	29	9
3	33	F	High school	Yes	S	27	11
4	33	M	College	Yes	S	27	11
5	20	F	Grade 9	No	S	26	12
6	29	M	Grade 10	Yes	S	26	12
7	29	F	Tertiary	Yes	T	31	7
8	28	F	Tertiary	Yes	S	35	5
9	29	M	Tertiary	Yes	S	35	5
10	30	F	Tertiary	Yes	S	30	8
11	42	F	Tertiary	Yes	S	31	7

Note: Married couples parenting one infant: (3 + 4); (5 + 6) and (8 + 9).

S, singleton; T, twin; GA, gestational age; NICU, neonatal intensive care unit; F, female; M, male.

In Table 1, all 11 parents who were approached agreed to participate in the study. All participants were married, and the ages ranged from 27 to 42 years. Out of eleven participants, three were male and eight were female; all parents had a high school diploma or higher, and only two were unemployed.

The gestational age of the premature babies at birth ranged between 26 and 35 weeks, and the babies were admitted for an average of 8.6 weeks in the NICU. All babies were discharged from the NICUs to their homes.

Regarding the characteristics of the pregnancies, two of the pregnancies were unplanned and one of the pregnancies was achieved with a fertility intervention.

The distances travelled to and from home to the NICU ranged from less than 15 km (two parents), 15 km – 30 km (four parents) to more than 30 km (five parents). As no accommodation for parents was provided at the hospitals during NICU admissions, this added to their stress. Parents also indicated that they received no formal orientation upon admission to the NICU, either verbally or in writing.

### Emerging themes, sub-themes and categories

Three themes emerged from the generated codes; each theme was developed into sub-themes and categories. The themes highlighted the lived parental experiences of their admission to the NICU, starting with the complicated pregnancy and the unexpected birth of their baby. Table 2 summarises the themes, sub-themes and categories that emerged from the interviews.

**TABLE 2:** Summary of themes, sub-themes and categories.

Themes	Sub-themes	Categories
1. Journey to NICU – Context of the pregnancy	1.1. Pregnancy experience	1.1.1. Planned or unplanned pregnancy 1.1.2. High-risk pregnancy 1.1.3. Emotionally complex pregnancy 1.1.4. Managing multiple responsibilities 1.1.5. Support during pregnancy
	1.2. Delivery experience	1.2.1. Birth plan 1.2.2. Self-blame for premature delivery 1.2.3. Concern for the infant 1.2.4. Concern for partner
2. Transition to parenthood in NICU – Parenting in an unnatural environment	2.1. Lived realities of the parents	2.1.1. The NICU environment 2.1.2. Positive experiences 2.1.3. Information sharing 2.1.4. Separation 2.1.5. Medical fragility of the infant 2.1.6. Managing multiple responsibilities 2.1.7. Cultural considerations
	2.2. Organisational challenges	2.2.1. Hospital failures and irritations 2.2.2. Internal architecture failures 2.2.3. Lack of accommodation
3. Coping with the emotional journey during a prolonged admission	3.1. Psychosocial challenges in the NICU	3.1.1. Rollercoaster of emotions 3.1.2. Parent worries 3.1.3. Perseverance and positivity
	3.2. Seeking support	3.2.1. Religious support 3.2.2. Partner support 3.2.3. Peer support 3.2.4. Professional support

NICU, neonatal intensive care unit.

### Theme 1: Journey to the neonatal intensive care unit

Theme 1 broadly describes the pregnancy journey that led to the admission of the preterm baby to the NICU. The range of emotional and physical stressors experienced by both mothers and fathers during pregnancy created emotional vulnerability in parents even before the admission of their premature baby to the NICU.

#### Sub-theme 1.1: Pregnancy experience

The first sub-theme introduces the medically complex pregnancies that were associated with increased distress regarding the health outcomes of both mother and baby. Most pregnancies, except one, were fraught with medical complications. The complications varied from gestational diabetes and pre-eclampsia to the need for a hysterectomy because of placental complications. One participant described her medically challenged pregnancy as:

‘So, my pregnancy was terrible. I had a placenta previa and then a placenta accreta. And then I got gestational diabetes, so I was on bedrest from 24 weeks, and then I was admitted at 28 weeks for bedrest in the hospital.’ (Participant 11, 30, female)

For the fathers, their experiences of the high-risk pregnancy were confounded by not only supporting their wives emotionally but also navigating added unexpected responsibilities in the home, whilst still navigating work responsibilities and doctor’s visits. This resulted in immense stress for the fathers:

‘From a father’s perspective or husband’s perspective … the emotions that you must deal with and the up and down swings with your partner, it’s tough to handle, let’s just be honest with that. I think also with my wife, she didn’t have a normal pregnancy as such; she had a lot more, I don’t want to say an emotional one.’ (Participant 4, 33, male)

‘In terms of the pregnancy, I think, obviously, there’s excitement and joy in the beginning. But as everyone thinks it’s all going to be sunshine and roses and rainbows, which isn’t.’ (Participant 4, 33, male)

‘I have to say the time before the NICU was more stressful.’ (Participant 9, 29, male)

#### Sub-theme 1.2: Delivery experience

Sub-theme two of the theme further depicts the baby’s unexpected birth. All the babies were delivered by emergency caesarean section (C-section). This was not necessarily anticipated nor desired by the parents as the method of delivery. However, medical circumstances and complications dictated the course of the delivery.

For the fathers, in particular, the delivery and the perceived responsibility for both their partner and the baby were overwhelming:

‘And then you know waiting for my wife to come out, sitting there for 40 minutes plus waiting, is she okay, is she not okay, if she’s not alive? So, all of that does work on you on your brain a bit.’ (Participant 4, 33, male)

Feelings of relief following the successful delivery of their child were tempered by the knowledge of the immediate medical risks and fragility of the newborn, which required admission to the NICU:

‘Your baby is going to die if you don’t deliver right now. And within like 10 minutes, they had a theatre ready for another C-section.’ (Participant 2, 27, female)

‘Once baby came out, it’s a sense of relief, but now the focus is to try and keep them alive because he was so premature at 27 weeks.’ (Participant 4, 33, male)

### Theme 2: Transition to parenthood in neonatal intensive care unit – Parenting in an unnatural environment

This theme describes the parenting experiences and the fundamental factors that shaped and influenced the transition to parenthood in the unnatural environment of the NICU. Two sub-themes were involved in this theme: (1) Parents’ lived realities and (2) organisational challenges.

There was a juxtaposition of positive and negative experiences that influenced the participants’ perceptions of parenting in the NICU.

#### Sub-theme 2.1: Lived realities of the parents

Navigating the NICU, two quotes paint a picture of the unknown environment:

‘And I remember the first time I walked in … they put a sign outside the NICU saying “Welcome to Premmiville” and I told my husband I wish you could change the sign to “Welcome to hell,” because this is hell for me.’ (Participant 2, 27, female)

‘The people that have never been in a NICU experience have no idea what actually goes on, it’s a different world – it’s crazy what goes on there … If you haven’t ever been in there, you have no idea.’ (Participant 10, 30, female)

Five central parental challenges emerged, underpinning the lived realities of the NICU: physical environment, lack of information sharing, physical separation, management of the medical fragility of their baby and management of additional personal and familial responsibilities. The following quotations best illustrate the five elements of the parents’ lived experiences.

Parenting in the NICU was influenced by the NICU environment, the effects of the numerous sounds and the presence of life-saving medical equipment:

‘And with all this going on, you still had all these sensors and alarms going off … I became so obsessed with it that the moment anything went a little funny, you start almost hyperventilating … so, for my side that was quite taxing as such, and they were tough days.’ (Participant 3, 33, female)

The parents’ experience of information sharing regarding NICU orientation and information was perceived as limited and insufficient. Only two parents had previous NICU experience with older siblings:

‘You know, and I think they’ve got the knowledge, but they’re not sharing it, because it’s such a normal environment for them. So, whatever is happening is logical for them. But it’s the most illogical thing for you.’ (Participant 1, 30, female)

Because of limited visiting hours, a lack of accommodation at the hospital and responsibilities at home, all these factors lead to physical separation between the baby and its parents:

‘I don’t wish that on anybody to leave your child and come home without your baby. And you wake up in the middle of the night, and you wonder if your child is okay. Because they’re not with you.’ (Participant 2, 27, female)

Challenges in managing their baby’s medical fragility, including unexpected changes in medical status and procedures being performed on their baby, caused emotional distress for the parents:

‘It’s better waiting outside then seeing them doing whatever they need to do. That was like the worst experience, I would say actually, from everything.’ (Participant 9, 29, male)

For fathers and parents with older siblings, managing multiple responsibilities was physically demanding:

‘Trying to balance to go see your child and the hospital and then still trying to run a normal household, you know, feeding dogs, looking after mommy, making sure that the washing is done.’ (Participant 9, 29, male)

The NICU experience was marked by both positive and negative experiences. The parents valued participating in care routines in the NICU and watching their babies reach developmental milestones:

‘I can’t remember anything else besides the new milestones that you reach, you know like getting to put clothes on her for the first time and that kind of thing.’ (Participant 10, 30, female)

Over and above these elements, the parents’ feelings of trust and respect for the medical staff engendered a positive experience:

‘It’s a different kind of nursing staff to your general nursing staff. They, they’ve got a special soul to them. They really do.’ (Participant 11, 42, female)

#### Sub-theme 2.2: Organisational challenges

The implication of organisational challenges - the physical structural failures, of not having a dedicated space for mothers to express breastmilk and lack of accommodation reduced privacy and created physical discomfort during the time parents spent in the NICU. The physical infrastructural challenges included the absence of an appropriate private room for expressing breastmilk and uncomfortable seating in the NICU for the mothers:

‘So now you’ve got to find and ask the unit manager where she can pump, and she said go and pump at home. I live half an hour away; I’m not going to drive home half an hour for my wife to pump and drive all the way back. There was a day she sat in the toilet and pumped, and to me, I’m like that’s not sanitary.’ (Participant 4, 33, male)

Because of the lack of accommodation for the parents, several parents were forced to travel long distances to visit their babies daily. The ensuing financial and time losses affected visitation and time spent with their babies:

‘I would spend a few hours, and then I would come home and then drive back, so I was probably back and forth three or four times a day ….’ (Participant 10, 33, female)

### Theme 3: Coping with the emotional journey during a prolonged admission

The prolonged NICU admission tested parental resilience because of the numerous psychosocial challenges experienced. The two sub-themes of theme 3 were informed by these challenges and the support the parents required to cope.

#### Sub-theme 3.1: Psychosocial challenges in the neonatal intensive care unit

All the parents vividly communicated their psychological and emotional journey during the NICU admission. They expressed emotions such as worry, vulnerability, fear and disappointment:

‘It’s a really taxing, emotional journey.’ (Participant 3, 33, female)

‘I mean, obviously it’s heartbreaking to go through.’ (Participant 10, 30, female)

With each day being different because of the nature of their baby’s medical fragility, it resulted in intense emotional experiences throughout the NICU admission:

‘But it was just such a rollercoaster.’ (Participant 1, mother of twins)

‘And obviously if something happens, like it just goes well and then there is a setback. Then it goes well again, so it’s kind of it’s an emotional rollercoaster.’ (Participant 8, 28, female)

#### Sub-theme 3.2: Seeking support

In adjusting to the abnormal environment of the NICU, the ramifications of prematurity, and the psychosocial consequences, parents needed to manage their daily physical and emotional needs by developing coping mechanisms and resilience. Resilience was noted among some parents as the admission period continued:

‘It’s not the same dad that walked in. Yeah, when he first walked in there, he couldn’t even spend ten seconds … Then it was five minutes. Then it was an hour.’ (Participant 3, 33, female)

Another coping mechanism was seeking support. The support varied, but in all cases it was informal. It was apparent that both mothers and fathers needed and benefited from spousal support, particularly emotional support:

‘So, I was coping better when my husband was there, and yeah, if he wasn’t there, then I was like sitting and crying and not coping and just feeling like I’m a bad mother.’ (Participant 8, 28, female)

‘But we pushed each other to still go irrespective of what we were going through or what stresses and pressures we had from a personal level and then, you know, from a relationship level supporting each other still is, was, massive. That was massive. Yeah, I can’t stretch the fact on it.’ (Participant 4, 33, male)

Overwhelmingly, parents expressed the benefits of peer support during their NICU admission. The support was mostly informal, not in an organised group setting:

‘I think meeting parents … that was sort of a nice experience, and having people that are going through the same thing with you helps because almost everyone else doesn’t really understand.’ (Participant 11, 42, female)

Varied levels of emotional support from the medical staff were offered in the NICUs. The parents (study participants) were admitted to different NICU facilities in Johannesburg, each offering varying levels of support. The nurses provided more situational support rather than structured, regular professional interventions. The support one participant received was limited to nursing support and none from trained mental health professionals:

‘When you’d have your emotional breakdown, there was always a nurse hugging you and telling you it was OK.’ (Participant 3, 33, female)

Whereas other parents did not feel supported by the medical staff in their NICU:

‘One thing I’d add was that in terms of emotional support, there wasn’t any.’ (Participant 11, 42, female)

## Discussion

The findings of this study contribute to the growing body of knowledge of parental experiences of the prolonged NICU hospitalisation of their premature babies within the South African private healthcare sector. Three overarching themes were revealed, detailing the psychological impact of high-risk pregnancies, the challenges of parental role acquisition in the NICU, and the reliance on informal support structures to navigate this challenging experience.

The first theme depicted the journey to the NICU experience by describing the parental difficulties following a high-risk pregnancy and the resultant early birth of their baby. All except one of the mothers encountered medical complications during the pregnancy, and all babies were delivered via unplanned C-section. This resulted in most of the participants describing their pregnancies and the delivery of a premature baby as extremely stressful events. Our findings resonate with the literature that the parental experience of a premature birth can be considered a definable traumatic event, with all the consequential ramifications of feelings of helplessness and fear (Malouf et al. 2022). During the antenatal period, the psychological and physiological processes of transitioning to parenthood begin (Ettenberger et al. 2021). A high-risk pregnancy can, however, disrupt this normal transitioning process and result in a decreased ability to cope, poorer well-being, and an increase in anxiety, depression, and stress (Williamson et al. 2023). Protective factors that mitigate these psychosocial stressors antenatally are twofold: (i) social support from friends and family and (ii) knowledge-based care and education from healthcare professionals (Williamson et al. 2023). The quality of obstetric care should be measured by the holistic management of pregnant women, ensuring appropriate and necessary culturally sensitive medical, social and psychological interventions (Alves, Cecatti & Souza 2021). Despite our participants all accessing private antenatal healthcare, no formal psychological support or education regarding a premature delivery was offered.

The second theme focused on the assumption of their parenting roles in the unnatural setting of the NICU. Parental reports of both positive and negative experiences shaped this theme.

Five central challenges that emerged underpin the difficulties of assuming the parental role in the NICU: the NICU’s physical environment, lack of information sharing, physical separation, managing their baby’s medical fragility, and managing additional personal and familial responsibilities. The noise and presence of medical equipment were identified by parents as barriers to fulfilling their parental role and are well described in the literature as adversely affecting parent–infant attachment and increasing parental stress (Siva et al. 2024). The parents in this study emphasised that information sharing and a structured NICU orientation would have helped them cope more effectively, particularly because none of them received written information about NICU procedures or the environment. This is contrary to recommendations that appropriate information sharing, particularly regarding medical care, procedures and NICU equipment, plays a two-fold role in reducing stress and changing the parental experience (Ginsberg et al. 2025; Yeomans et al. 2026). Despite the physical separation from their babies, resulting from either their baby’s medical fragility or having to leave their baby in the NICU, whilst travelling long distances to and from the NICU, the parents described their participation in caregiving activities as symbolic of autonomy, trust and physical connection, and as facilitating parental role assumption. The literature strongly supports parental participation in care routines as a means of fostering the transition to parenthood in the NICU (Kutahyalioglu & Scafide 2023).

The third theme of this study highlighted the psychosocial challenges encountered by the parents during the prolonged hospitalisation of their premature babies in the NICU, as well as the coping strategies they employed. Describing the psychological journey of the NICU experience as a ‘rollercoaster of emotions’ is not a concept unique to this study, but has indeed been described in previous research (Ginsberg et al. 2025; Nukpezah & Atanuriba 2025).

Across a spectrum of countries, independent of socio-economic status, parental emotional distress is a prominent experience during NICU admission (Ginsberg et al. 2025; Hemle Jerntorp, Sivberg & Lundqvist 2021; Nukpezah & Atanuriba 2025). Parents in the current research articulated their emotional and psychological distress, irrespective of which hospital they were admitted to, highlighting that this theme is universal and not specific to context or hospital-dependent. In unpacking the mothers’ and fathers’ emotions, different emotions associated with specific stressors and events in the NICU emerged. Fear, shock and worry were often associated with the medical fragility of the baby, the appearance of the baby, and the possible short- and long-term consequences of prematurity. These emotions are similarly supported by research (Siva et al. 2024; Steyn et al. 2017).

In coping with these challenges, parents relied on informal support from spouses, peers in the NICU, and, intermittently, from nursing staff. Notably, none of the participants described structured professional support from psychologists, psychiatrists or social workers. This absence contrasts sharply with international research emphasising the importance of the presence of these professionals in the NICU (Bloyd et al. 2022; Osborne et al. 2025).

Similarly, no formal educational support was offered to the parents before the delivery of their premature baby, despite having high-risk pregnancies, nor during the prolonged NICU stay, as discussed under theme two. Educational support in the NICU should ideally be individualised based on maternal and infant-related factors, specifically the medical challenges of the infant and the duration of the NICU stay (Lubbe & Donald 2026). These findings are concerning when considering the supposition that private South African healthcare providers provide good, holistic care to the maternal-infant dyad, as they are better resourced.

The benefits of implementing FCC practices are well documented, including improved infant weight gain, as well as parental benefits such as reduced stress, greater satisfaction and improved clinical knowledge (Lubbe, Kruger & Donald 2026; Sivanandan et al. 2021). Integrating structured psychosocial and educational support into NICU care, both in the public and private healthcare sectors, would therefore not only address the emotional distress and innate need to assume the parental role, but also strengthen attachment and improve long-term outcomes for families navigating the challenges of prematurity that require prolonged hospitalisation. The lack of comprehensive psychosocial care and educational support in private-sector NICUs in South Africa, identified in this study, represents a critical gap in neonatal care and service delivery.

### Implications for future practice

This research highlights, discusses and reinforces two common themes that have previously emerged in the literature: the challenges of emotional distress parents experience during the admission period in an NICU and the difficulties in the process of achieving the parental role when parenting a premature baby in the NICU. Compounding these challenges, limited access and exposure to professional psychosocial counselling arose as an additional important theme.

Emerging from the narratives of the 11 interviews, it became apparent that none of the participants received or was referred to structured psychosocial support during their high-risk pregnancy or NICU admission period.

This information, however, cannot be taken as an indication that no such services exist in the four private hospital settings where the study participants received NICU care. Neither can it be assumed to reflect all private healthcare settings in Johannesburg. Identifying these context-specific experiences of parents in South African NICU settings is an important contribution to informing policy, promoting FCC in NICU environments and ensuring that the multidisciplinary team provides appropriate education on how to care for the parents of a hospitalised neonate.

There remains a need for South African NICU facilities to develop context-specific guidelines and best-practice standards for psychosocial support for NICU parents.

### Strengths and limitations

This research added to the current understanding of parents’ lived experiences during the admission of their preterm babies to a private NICU in Johannesburg. Including parents who attend four different facilities highlights the similarities in their experiences. However, the findings of this research cannot be generalised to all private NICU settings in South Africa.

Despite purposive sampling, the parents included in the study did not reflect the full diversity of the South African population accessing private healthcare in Johannesburg. Choosing the private healthcare sector rather than the public sector further limited the diversity of the parents interviewed and of NICUs.

## Conclusion

Having a baby admitted to the NICU was described as emotionally overwhelming, and many parents found it difficult to establish their role and confidence as caregivers while their baby remained in the unit. An important concern that emerged was the absence of psychosocial support, as none of the eleven participants reported receiving formal counselling or being referred to support services during this period. Although the study focused on a small group of parents from private NICU settings and the findings cannot be applied to all South African contexts, it offers valuable insight into an area that has received limited attention. These accounts highlight the importance of recognising parents’ emotional needs, strengthening family-centred care practices, and developing contextually appropriate guidelines to support families throughout the NICU journey.
